# Independent association between prostate-specific antigen nadir and PSA progression-free survival in first-line abiraterone acetate treatment in castration-resistant prostate cancer patients: a pilot study

**DOI:** 10.3389/fonc.2024.1348324

**Published:** 2024-06-04

**Authors:** Hong Du, Wenjuan Xie, Wenqiang Chen, Yu Wang, Yong Liao, Mingxing Qiu, Jun Li

**Affiliations:** ^1^ Department of Urology, Sichuan Provincial People’s Hospital, University of Electronic Science and Technology of China, Chengdu, China; ^2^ Human Anatomy and Tissue Embryo Experiment Center, Chengdu Medical College, Chengdu, China

**Keywords:** prostate-specific antigen (PSA) decline and kinetics, progression free survival, association, generalized additive model, subgroup analysis

## Abstract

**Background:**

There is limited evidence regarding the correlation between prostate-specific antigen (PSA) kinetics and clinical outcomes. Therefore, after regulating other covariates, we studied patients with castration-resistant prostate cancer who received abiraterone acetate as the first-line treatment. In this study, we investigated whether time to PSA nadir was independently associated with PSA progression-free survival (PFS).

**Methods:**

As a retrospective cohort study, this study contained a total of 77 castration-resistant prostate cancer patients who received abiraterone acetate from October 2015 to April 2021 in a Chinese hospital. The dependent variable was PSA-PFS. The objective independent variable was time to PSA nadir (TTPN). Covariates involved in this study included age, duration of androgen deprivation therapy (ADT), PSA level at baseline, time of 50% PSA decline, time of PSA decline to nadir, Gleason score, bone metastasis, previous treatment, PSA decline <50% in 3 months, PSA to nadir in 3 months, PSA decline <90%, PSA decline <0.2 ng/mL, and PSA flare.

**Results:**

For the 77 subjects, their mean age was 72.70 ± 8.08 years. Fully calibrated linear regression findings indicated that PSA decline and kinetics were positively associated with PFS (months) after adjusting confounders (β = 0.77, 95% CI: 0.11–1.44). A non-linear relationship was not detected between PSA decline or PSA kinetics and progression-free survival.

**Conclusion:**

According to the data of this study, there was a correlation between early PSA changes and patients treated with abiraterone acetate.

## 1 Introduction

Prostate cancer (PCa) is the second most common cause of cancer-related death and the most prevalent malignancy in men. It is considered that an aggressive disease is recently diagnosed metastatic castration-sensitive prostate cancer (mCSPC) that quickly grows to a castration-resistant condition and poor viability (1). With the advent of updated androgen receptor (AR)-targeted drugs, the metastatic castration-resistant prostate cancer (mCRPC) treatment tactics have been influenced significantly. It has been demonstrated that enzalutamide and abiraterone acetate are AR-targeted drugs that are helpful to patient survival (1). There is a close correlation between disease burden and prognosis in the mCRPC setting with AR regulatory protein and serum prostate-specific antigen (PSA) levels (2, 3). With the suppression of the AR pathway, PSA levels decreased greatly. Based on AR signaling, it is known that CRPC cells remain active in castrated serum testosterone levels, and CRPC is thought to play a significant function in developing hormones from CRPC. Therefore, it is likely for CRPC that the androgen–AR axis is the best treatment target (4). Indeed, mCRPC patients treated with abiraterone acetate (AA) and enzalutamide (Enz) (both targeting AR signaling) have significantly improved overall survival (OS) in chemotherapy-naïve and refractory patients, according to the latest performed major randomized clinical experiment (5–9). Nevertheless, OS in patients with mCRPC can be prolonged by introducing additional treatment regimens (4, 10). Therefore, patients who initially respond to androgen receptor axis-targeted (ARAT) drugs but may experience rapid disease progression need to be promptly identified. Giving an optional formulation at an optimal time minimizes disease symptom exacerbation. There are a number of useful metrics for assessing response to many systemic therapies in patients with advanced PC, such as PSA kinetics, PSA nadir, PSA progression, PSA velocity, PSA doubling time, and time to PSA nadir (TTPN) (11–13). The TTPN prognostic significance in PC patients, in particular, has been greatly described (14–22). To be specific, faster TTPN associated with shorter OS in hormone-sensitive PC patients receiving androgen deprivation therapy (ADT) was presented by Choueiri et al. (15). Thomas et al. (17) found that TTPN at 16 weeks after initiation of docetaxel was a separate predictor of shorter progression-free survival in patients with mCRPC. There was a remarkable impact of the presence of PSA kinetics on clinical outcomes in patients with mCRPC receiving either drug based on the fact that the AR signaling pathway may be disrupted by the mechanism of action of AA and Enz (4). However, the TTPN prognostic significance is still inadequate in mCRPC patients treated with AA or Enz. Taking these outcomes into account, 297 docetaxel-naïve Japanese mCRPC patients were totally evaluated for TTPN prognostic significance. These patients achieved a degree of PSA reduction following AA or Enz treatment.

Monitoring response to treatment is usually performed using a PSA decrease. Nevertheless, at present, especially in patients receiving abiraterone acetate, it has not been confirmed as a progression-free survival indicator. The outcomes of earlier research have been inadequate, however. Several studies have shown that there is a good prognosis related to early PSA in mCRPC response to AR-targeted drugs. Conversely, there is a correlation between elevated PSA and a higher likelihood of biochemical development. These studies differed in target population, design, and data analysis. Further research is still required. Based on earlier studies, there is a correlation between PSA development and shorter survival under the PCWG2 standard. Compared with a 50% PSA drop, there was a stronger correlation between a 30% PSA drop and survival. A retrospective cohort study in mCSPC patients treated with abiraterone acetate was performed to identify the relationship between PSA decline/kinetics and progression-free survival.

## 2 Participants and methods

### 2.1 Study design and study population

This retrospective cohort study included a total of 77 Chinese men with mCRPC who were treated with AA as initial therapy between October 2015 and April 2021 in a routine clinical setting and subsequently finished the follow-up at Sichuan Provincial People’s Hospital. Data were obtained from the electronic medical record system of the Urology Department. Each participant provided written informed consent before data collection. The institutional research ethics committee approved the design of this study.

Eligible patients who had histopathologically diagnosed prostate cancer and chemotherapy-naïve disease that progressed during androgen deprivation therapy were included. To note, PSA or X-ray progression in patients with serum testosterone levels of 50 ng/dL is a progressive disease of primary ADT, suggesting the development of CRPC. Additional inclusion criteria are as follows: with Eastern Cooperative Oncology Group performance status of ≤1 and without any metastases other than bone metastases and regional lymph nodes without abnormality in liver function, kidney function, and blood routine tests. Men who had received previous chemotherapy, radiotherapy, or surgery and those who had received other novel ARAT agents such as enzalutamide were excluded. Meanwhile, patients with known allergies or contraindications to AA were not enrolled.

### 2.2 Treatment and follow-up

Patients received abiraterone acetate and prednisone 1,000 mg once daily and prednisone 5 mg twice daily. When treatment-associated adverse events occurred, dose modification was permitted by the physicians. Patients were given AA before disease development (as judged by the same definition as primary ADT) or other cancer development events occurred. Patients were followed up monthly, including rechecking serum PSA levels; imaging examinations such as bone scans and whole abdomen enhanced CT were reviewed every 3 months to check the patients’ treatment status. The cutoff date for participants’ follow-up was April 1, 2021. Follow-up data were documented until the last follow-up. The follow-up interval was 1 month, and follow-up data in the hospital’s electronic medical record system were reserved. PSA values and radiological assessments were included in the monitoring measures at each visit.

### 2.3 Variables

We obtained patient demographic and clinicopathological data at baseline and PSA kinetics at follow-up after the introduction of AA from each patient’s medical record. This study evaluated the Eastern Cooperative Oncology Group’s performance status (ECOG PS). We used radionuclide bone scans for radiological assessment and computed tomography to measure metastatic disease’s amount and location. The serum values of PSA were measured before the AA treatment initiation by automated chemiluminescence immunoassay (CLIA) and at every visit and recorded as a continuous variable.

The following definitions related to PSA kinetics were used in this study: TTPN (time from the onset of action of any agent to PSA nadir), PSA nadir (lowest PSA achieved during dosing), and PSA response (PSA > 50% decrease from baseline).

The ultimate result variable (continuous variable) was gained by us based on published studies. An increase in PSA levels greater than 25% of the nadir and/or an increase of ≥2 ng/mL is referred to as PSA progression. These criteria are based on recommendations from the Prostate Cancer Working Group 2 (PCWG2) (23).

### 2.4 Statistical analysis

Frequencies or percentages represent categorical variables. Differences in characteristics among different TTPN groups (tertile) were compared using the unpaired t-test and chi-square test. Univariate and multivariate linear regressions were employed to classify the relationship between TTPN and PSA-PFS. To further evaluate the association between the TTPN and the endpoint of our study, for each outcome variable, three models were formed without covariate adjustment, including Adjusted Model I with age, Gleason score, bone metastasis, and ECOG performance status; and Adjusted Model II with all the baseline characteristics including age, Gleason score, bone metastasis, ECOG performance status, duration of ADT, and baseline PSA. To determine the PSA progression-free survival in different TTPN groups (dichotomous and tertile), the Kaplan–Meier method was used for survival analysis. Also, tests determine significance by log rank. Subgroup analysis and forest plot were performed as a sensitivity test.

R-4.1.2 statistical package analyzed the data from all studies (http://www.R-project.org, The R Foundation). This study used a two-tailed test. Differences were considered statistically significant at p < 0.05.

## 3 Results

### 3.1 Participant characteristics

**Figure 1:**
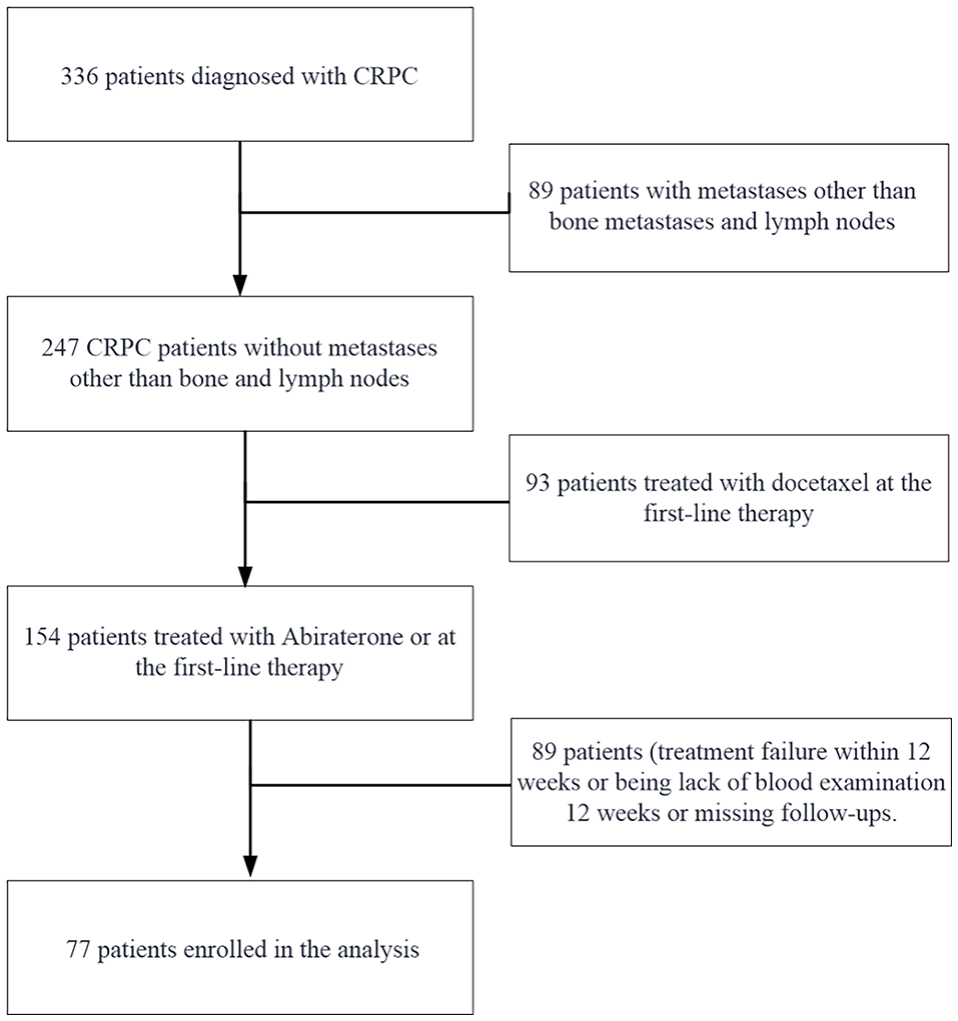
Flowchart of the study population selection.

Ultimately, 77 participants were selected for data analysis (see **Figure 1** for a flowchart). In the flowchart for selecting the study population, patients who progressed within 12 weeks were excluded from the final analysis if they experienced treatment failure or lacked blood test results during that time period, which resulted in incomplete data or no conclusions about the study. Their exclusion ensured a more robust and reliable analysis of the remaining patient cohort. For the final analysis population, the average follow-up duration was 14.69 ± 5.14 months.

We summarized the basic characteristics of the 77 enrolled men in each group (Q1, Q2, and Q3) according to the mean TTPN in **Table 1**. In general, the mean age for participants at the diagnosis of cancer was 72.71 ± 8.08 years. The highest baseline PSA values occurred in participants in the highest TTPN (Q3) group but with no statistically significant differences. Meanwhile, among other baseline characteristic differences, there was no statistical significance, including Gleason score, bone metastasis, ECOG performance status, and duration of ADT (p-values >0.05). After receiving abiraterone acetate, the median PSA-PFS of these Chinese men was 14.69 ± 5.14 months. Patients with a longer duration of TTPN (Q3) had better PSA-PFS. There were significant differences in PSA kinetics variables including the time to PSA reduction >50%, PSA declined to 0.2 ng/mL, and the PSA flare between different TTPN groups.

**Table 1:** Characteristics of patients with docetaxel-naïve, metastatic castration-resistant prostate cancer receiving AA according to the TTPN.

	Time to prostate-specific antigen (PSA) nadir (TTPN)			p-Value
	Q1 (2.87–4.17)	Q2 (4.93–6.90)	Q3 (6.93–14.13)	
Total, n	24	27	26	
TTPN	3.47 ± 0.50	5.62 ± 0.64	9.50 ± 2.30	<0.001
Age (years)	74.00 ± 8.16	71.52 ± 7.40	72.77 ± 8.79	0.555
Gleason score, %				0.183
7	4 (16.67)	1 (3.70)	3 (11.54)	
8	7 (29.17)	16 (59.26)	14 (53.85)	
9	13 (54.17)	10 (37.04)	9 (34.62)	
Bone metastasis,%				0.065
No	2 (8.33)	3 (11.11)	8 (30.77)	
Yes	22 (91.67)	24 (88.89)	18 (69.23)	
ECOG performance status, %				0.690
0	16 (66.67)	18 (75.00)	16 (64.00)	
1	8 (33.33)	6 (25.00)	9 (36.00)	
Duration of ADT, months	21.65 ± 9.35	19.17 ± 9.72	20.75 ± 10.58	0.662
PSA-PFS, months	11.18 ± 4.23	14.58 ± 4.75	18.04 ± 4.08	<0.001
Baseline PSA (ng/mL)	78.11 ± 92.43	73.43 ± 92.22	70.55 ± 115.51	0.965
Time to PSA reduction >50%, months	2.00 ± 0.95	3.09 ± 1.54	4.15 ± 2.05	<0.001
PSA reduction >50% in 3 months				<0.001
No	2 (8.33)	11 (40.74)	16 (61.54)	
Yes	22 (91.67)	16 (59.26)	10 (38.46)	
PSA reduction >90%				0.166
No	9 (37.50)	15 (55.56)	8 (30.77)	
Yes	15 (62.50)	12 (44.44)	18 (69.23)	
TTPN in 6 months				<0.001
No	0 (0.00)	4 (14.81)	26 (100.00)	
Yes	24 (100.00)	23 (85.19)	0 (0.00)	
PSA declined to 0.2 ng/mL				0.905
No	16 (66.67)	18 (66.67)	16 (61.54)	
Yes	8 (33.33)	9 (33.33)	10 (38.46)	
PSA flare				0.042
No	21 (87.50)	16 (59.26)	15 (57.69)	
Yes	3 (12.50)	11 (40.74)	11 (42.31)	

AA, abiraterone acetate; ADT, androgen deprivation therapy; PSA-PFS, prostate-specific antigen progression-free survival; ECOG, Eastern Cooperative Oncology Group.

### 3.2 Participant characteristics

**Figure 2:**
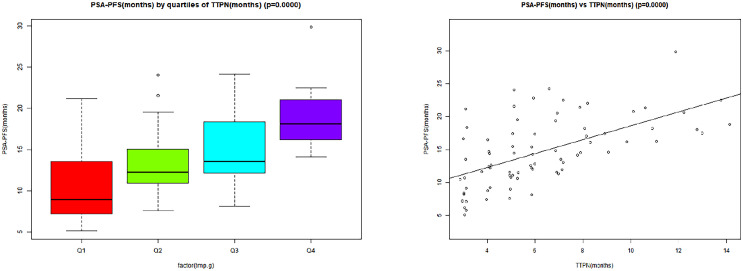
The linear association between TTPN and PSA-PFS. TTPN, time to prostate-specific antigen nadir; PSA-PFS, prostate-specific antigen progression-free survival.

**Table 2** presents the findings of univariate and multivariate analyses in order to assess the association of many clinicopathological factors with PSA-PFS. By univariate linear regression, we found that baseline variables were not associated with PSA-PFS. It could be seen from the univariate linear regression that there was no correlation between PSA-PFS and baseline variables. This study showed that TTPN in 6 months, TTPN in 3 months, and TTPN were positively associated with PSA-PFS. Univariate analysis determined other great PSA-PFS predictors: PSA reduction >90%, time to PSA reduction >50%, PSA declined to 0.2 ng/mL, and PSA flare. By multivariate analysis, TTPN and TTPN in 6 months were the most significant variables for the PSA-PFS (β = 0.67, 95% CI: 0.09, 1.26, p = 0.027; OR 0.67, 95% CI: 0.09, 1.26, p = 0.027). As shown in **Figure 2**, there was a tendency for better PSA-PFS in the TTPN quartile group of the higher mean value. The scatter plot also shows a clear linear relationship between TTPN and PSA-PFS.

**Table 2:** Univariate and multivariate analyses of associations between various parameters and PSA-PFS in mCRPC patients receiving AA.

Variables	Univariate analysis		Multivariate analysis	
	β/OR (95% CI)	p-Value	β/OR (95% CI)	p-Value
Age (years)	−0.03 (−0.17, 0.11)	0.673	–	–
Gleason score			–	–
7	0			
8	−0.85 (−4.82, 3.12)	0.677	–	–
9	−0.96 (−4.99, 3.07)	0.641	–	–
Bone metastasis				
No	0			
Yes	−1.77 (−4.83, 1.29)	0.260	–	–
ECOG performance status				
No	0			
Yes	−1.88 (−4.39, 0.64)	0.148	–	–
Duration of ADT	0.04 (−0.08, 0.16)	0.531	–	–
Baseline PSA	0.00 (−0.01, 0.01)	0.844	0.00 (−0.01, 0.01)	0.6399
Time to PSA declined >50%, months	0.80 (0.18, 1.42)	0.014	0.08 (−0.89, 1.06)	0.8653
PSA reduction >50% in 3 months				
No	0		0	
Yes	−3.41 (−5.66, −1.15)	0.004	−1.52 (−5.10, 2.05)	0.4062
PSA reduction >90%				
No	0		0	
Yes	4.13 (1.98, 6.28)	0.000	3.00 (0.61, 5.38)	0.0162
TTPN	1.05 (0.72, 1.38)	<0.001	0.67 (0.09, 1.26)	0.0270
TTPN in 6 months				
No	0		0	
Yes	−5.38 (−7.41, −3.34)	<0.001	−1.12 (−4.34, 2.11)	0.4992
PSA declined to 0.2 ng/mL				
No	0		0	
Yes	3.51 (1.22, 5.79)	0.004	1.29 (−1.18, 3.76)	0.3096
PSA flare				
No	0		0	
Yes	1.91 (−0.52, 4.34)	0.127	−0.81 (−3.39, 1.76)	0.5385

PSA-PFS, prostate-specific antigen progression-free survival; mCRPC, metastatic castration-resistant prostate cancer; AA, abiraterone acetate; ECOG, Eastern Cooperative Oncology Group; ADT, androgen deprivation therapy.

### 3.3 Results of unadjusted and adjusted linear regression

To identify the independent impacts of TTPN on PSA-PFS (univariate and multivariate linear regression), three models were formed in this study. **Table 3** presents confidence intervals and effect sizes. The difference in the model-based effect size of one unit TTPN was considered to be positively associated with PSA-PFS in the unadjusted model (crude model). PSA-PFS was used as the outcome variable, and the impacts of multiple confounding variables could be incorporated into the other two regression models simultaneously.

**Table 3:** Associations between TTPN and PSA-PFS in mCRPC patients receiving AA (Adjusted Model I adjusted for age, Gleason score, bone metastasis, and ECOG performance status; Adjusted Model II adjusted for age, Gleason score, bone metastasis, ECOG performance status, duration of ADT, and baseline PSA).

Variables	Crude model	Adjusted Model I	Adjusted Model I
TTPN	1.05 (0.72, 1.38) <0.001	1.10 (0.77, 1.43) <0.001	1.10 (0.76, 1.45) <0.001
TTPN dichotomous			
Low	0	0	0
High	4.88 (2.86, 6.91) <0.001	5.51 (3.45, 7.57) <0.001	5.75 (3.65, 7.85) <0.001
TTPN tertile			
Low	0	0	0
Middle	3.40 (1.00, 5.81) 0.007	3.72 (1.21, 6.24) 0.005	3.97 (1.34, 6.59) 0.004
High	6.86 (4.44, 9.29) <0.001	7.58 (5.11, 10.05) <0.001	7.79 (5.26, 10.31) <0.001

TTPN, time to prostate-specific antigen nadir; PSA-PFS, prostate-specific antigen progression-free survival; mCRPC, metastatic castration-resistant prostate cancer; AA, abiraterone acetate; ECOG, Eastern Cooperative Oncology Group; ADT, androgen deprivation therapy.

In the minimum-adjusted model (Adjusted Model I) and the fully adjusted model (Adjusted Model I) (adjusted all covariates at baseline), TTPN was the independent factor for PSA-PFS in Adjusted Model I (βadj 1.10 per month increase, 95% CI: 0.77–1.43; p ≤ 0.001) and Adjusted Model II (βadj 1.10 per month increase, 95% CI: 0.76–1.45; p ≤ 0.001).

For sensitivity analysis, we transformed TTPN from a continuous variable to a dichotomous categorical variable and a tertiary categorical variable. For sensitivity analysis, we converted the TTPN from a continuous variable to a dichotomous categorical variable and tertile categorical variable. Notably, it also demonstrated a similar overall relationship between TTPN and PSA-PFS.

### 3.4 The progression-free survival and subgroup analyses

**Figure 3:**
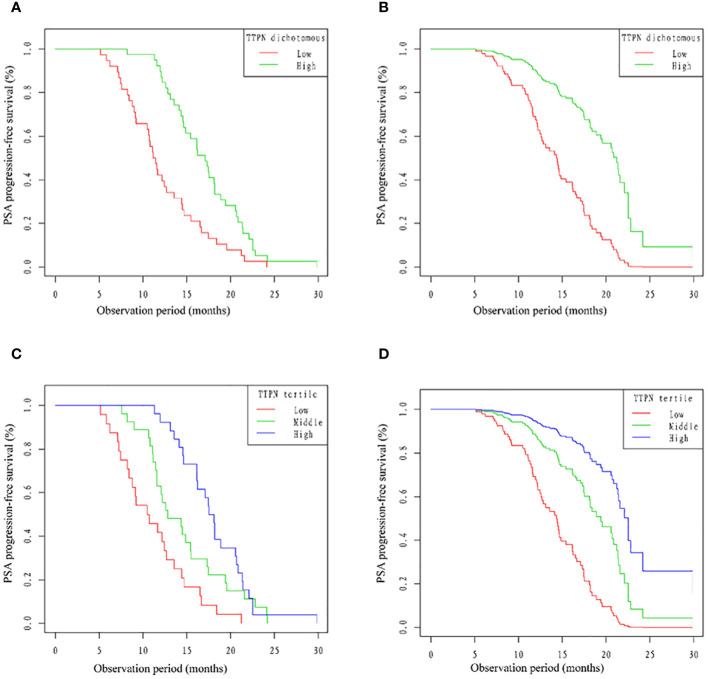
Kaplan–Meier curves of PSA-PFS and cardiovascular mortality stratified by different TTPN groups. PSA-PFS, prostate-specific antigen progression-free survival; TTPN, time to prostate-specific antigen nadir.

As shown in **Figure 3A**, compared with the high TTPN group, the median time to PSA progression was greatly higher in the low TTPN group patients after transforming TTPN from a continuous variable to a dichotomous categorical variable. After adjusting for confounders, such an effect was still stable (**Figure 3B**). After converting the TTPN, patients in TTPN groups presented a better PSA-PFS, especially after adjusting for the confounders (**Figures 3C, D**).

**Figure 4:**
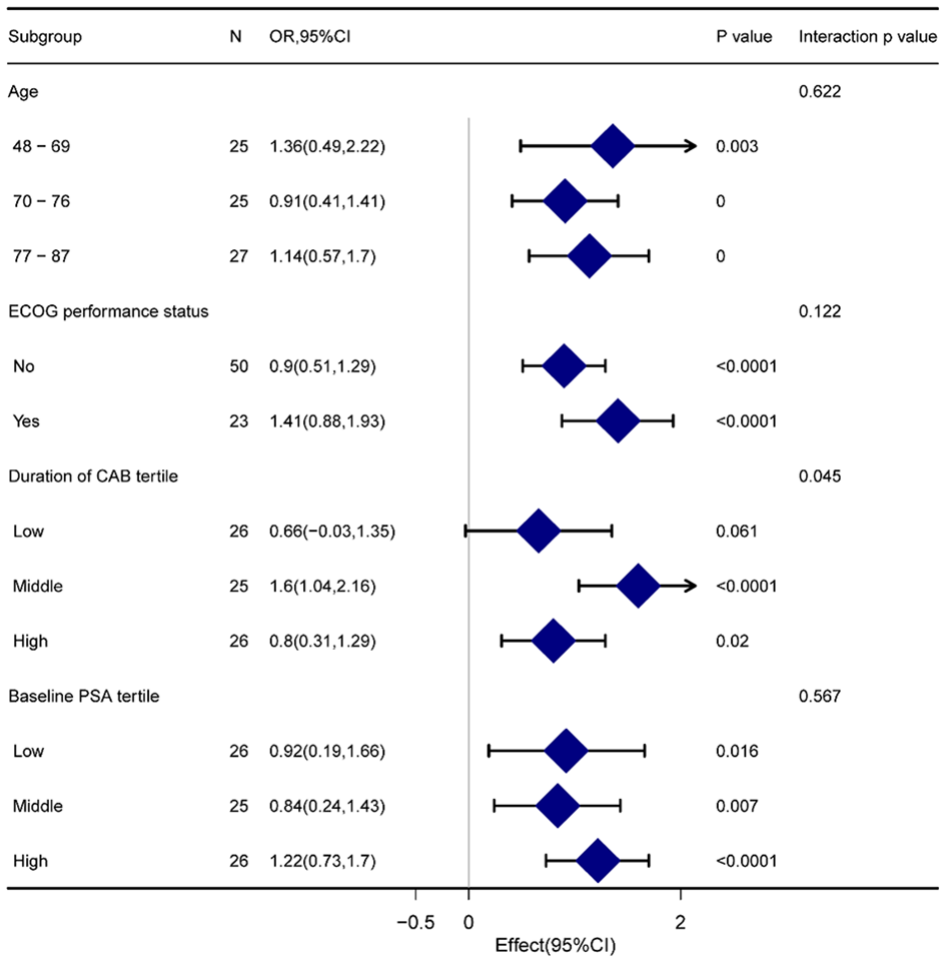
Forest plot for presenting the association between TTPN and PSA-PFS in different subgroups. TTPN, time to prostate-specific antigen nadir; PSA-PFS, prostate-specific antigen progression-free survival.

Age, ECOG performance, duration of ADT, and as a stratification variable were used. Trends in the effect size of the baseline PSA status variable were observed (**Figure 4**). Just a small number of interactions were noted in the group with low ADT duration. Except for this, all the p-values for each subgroup were over 0.05. Meanwhile, there was no linear or propensity change with the stratification variable (all p-values for interaction >0.05).

## 4 Discussion

This study showed that the time to PSA nadir was significantly faster in the Enz group than in the AA group, as the mean TTPN in the AA and Enz groups was approximately 19 and 14 weeks, respectively. TTPN in chemotherapy-resistant mCRPC patients treated with AA or Enz was assessed by Caffo et al. (24). Results presented a median TTPN of 15.5 months for the AA group and 7.0 months for the Enz group. Additionally, compared with patients in the AA group whose TTPN R was 19 weeks, TTPN 419 weeks patients may have more greatly favorable characteristics, although several parameters of TTPN in the Enz group lacked significant differences.

It can be found that after introducing AA, the PSA of mCRPC patients could not be decreased rapidly. However, PSA is expected to decrease further, especially in patients in good condition. Then, the TTPN influence on the prognostic outcome of mCRPC patients treated with AA or Enz was studied. PSA response rate or PSA-PFS in the Enz group was not greatly affected by TTPN. However, in the Enz group, the PSA response rate and PSA-PFS appeared to be greatly higher in patients with TTPN 419 weeks than in patients with TTPN R 19 weeks. Likewise, longer TTPN was greatly related to better OS in mCRPC patients who did not receive chemotherapy and who received AA before treatment, according to Xu et al. (20). Resolving the mechanisms for the various TTPN prognostic influences on the AA and Enz groups was hard. However, we could explain the association between TTPN and disease control in the AA (but not the Enz group) group based on the diverse action principles between AA and Enz. AA rapidly eradicated endocrine androgen dependence and AR dependence, at least in part. CRPC cells could provide more suitable androgen-independent and AR-dependent CRPC cells. An environment grown through AA could provide a more suitable environment for growth. Androgen-independent and AR-dependent CRPC cells, characterized by an anti-AA phenotype, and Enz could theoretically be therapeutically active in both types of CRPC cells.

The parameters adopted to evaluate the prognosis of mCRPC patients receiving novel AR axis-targeted agents are of interest. This is because of the lack of reliable model systems to evaluate the treatment of chemotherapy-naïve mCRPC with these agents particularly during pre-chemotherapy (10, 25). In the Enz group, the series showed that PSA-PFS was affected by age and ECOG PS, while in the AA group, the series showed that PSA-PFS was affected independently by ECOG PS and TTPN. Until now, for individual patients with mCRPC who have not received docetaxel, we still cannot use the prediction parameters that drive the choice between AA and Enz. Therefore, is very beneficial in identifying appropriate novel ARAT agents after primary ADT failure. In addition, in routine clinical practice, shorter TTPN is recommended as one of the parameters for introducing alternative medicines to docetaxel-naïve mCRPC patients treated with AA rather than Enz.

In this study, some deficiencies are discussed here. First, as a retrospective study, because of the observation period of this study, it is difficult to summarize firm conclusions on prognostic issues. However, this is one of the largest reported series for mCRPC patients receiving novel ARAT drugs prior to docetaxel. Therefore, an extended study period to identify the influence of TTPN on OS prospectively is warranted. Then, chemotherapy-naïve mCRPC patients were only covered in this study. In order to more fully understand the TTPN prognostic significance after treatment with ARAT agents excluded, findings in chemotherapy-resistant mCRPC patients should also be analyzed. Therefore, the overall cohort of mCRPC cannot use the current findings. Last but not least, gaining some significant data from all included patients was hard since it was a retrospective study that was performed outside of clinical trials.

All in all, it could be proven that in Japanese mCRPC patients not treated with docetaxel, a longer time to PSA nadir after initiation of treatment with AA was shown to be an independent predictor of favorable PSA-PFS. As a result, a helpful parameter to determine mCRPC patients may be a shorter TTPN. These patients at first responded to AA prior to chemotherapy but may recrudesce quickly.

## Data availability statement

The datasets presented in this study can be found in online repositories. The names of the repository/repositories and accession number(s) can be found in the article/supplementary material.

## Ethics statement

The studies involving humans were approved by department of Urology, Sichuan Provincial People’s Hospital, University of Electronic Science and Technology of China, Chengdu, China. The studies were conducted in accordance with the local legislation and institutional requirements. The participants provided their written informed consent to participate in this study. Written informed consent was obtained from the individual(s) for the publication of any potentially identifiable images or data included in this article.

## Author contributions

HD: Conceptualization, Data curation, Formal analysis, Investigation, Methodology, Resources, Software, Supervision, Writing – original draft, Writing – review & editing. WX: Investigation, Methodology, Software, Supervision, Writing – original draft, Writing – review & editing. WC: Conceptualization, Formal analysis, Investigation, Methodology, Software, Writing – original draft, Writing – review & editing. YW: Conceptualization, Data curation, Investigation, Methodology, Project administration, Software, Supervision, Writing – original draft, Writing – review & editing. YL: Conceptualization, Data curation, Investigation, Methodology, Project administration, Software, Writing – original draft, Writing – review & editing. MQ: Conceptualization, Data curation, Formal analysis, Investigation, Methodology, Project administration, Resources, Software, Supervision, Writing – original draft, Writing – review & editing. JL: Data curation, Formal analysis, Investigation, Project administration, Resources, Writing – original draft, Writing – review & editing.
